# Consumers’ Willingness to Pay for Organic Foods in China: Bibliometric Review for an Emerging Literature

**DOI:** 10.3390/ijerph16101713

**Published:** 2019-05-16

**Authors:** Rui Li, Hsiu-Yu Lee, Yu-Ting Lin, Chih-Wei Liu, Prony F. Tsai

**Affiliations:** 1 Department of Food Quality and Safety, FoShan University, XianXi Lakeside, DaLi, NanHai District, Foshan 528231, China; 2 South China Food Safety Research Center, Foshan 528225, China; 3 Department of Business Administration, Cheng Shiu University, Kaohsiung 83347, Taiwan; leehsiuyu7533967@outlook.com (H.-Y.L.); maniera01@gmail.com (C.-W.L.); tsaifs@gcloud.csu.edu.tw (P.F.T.); 4 Department of Food & Beverage Management, Cheng Shiu University; Kaohsiung 83347, Taiwan; k3912@gcloud.csu.edu.tw; 5 Super Micro Mass Research and Technology Center, Cheng Shiu University, Kaohsiung 83347, Taiwan; 6 Center for Environmental Toxin and Emerging-Contaminant Research, Cheng Shiu University, Kaohsiung 83347, Taiwan

**Keywords:** willingness to pay (WTP), organic foods, China, Bibliometrics

## Abstract

We conducted a bibliometric review on a small but promising body of literature on consumers’ willingness to pay for organic foods in China. Results found that consumers’ health consciousness, individual norms, consumer knowledge, food safety, environmental concerns, animal welfare, and purchasing power are major influencing factors for willingness to pay for organic foods in China. Notably, most research methods utilized are quantitative methods, leading us to call for the adoption of more qualitative, review, or mixed-methods. These findings increase our understanding of the knowledge structure of this emerging context-specific literature.

## 1. Introduction

Organic food has proposed both opportunities and challenges for market consumers (and other stakeholders) in all of the world, especially in developing economies. There are numerous benefits that are associated with organic food; this includes the fact that it is better for the environment, richer in certain nutrients, healthier, safer, and good for the welfare of animals and future sustainability. Also, organic agriculture is gaining enormous popularity regarding providing food and income.

Willer and Lernoud [1] indicated that the total area in Asia dedicated to organic agriculture was about 4.9 million hectares of organic agricultural land in 2016, whereby the leading countries were China (2.28 million hectares) and India (almost 1.2 million hectares). Therefore, the rest of the global countries are left to share the 20%, which is shocking and raises concern on what influences the willingness of consumers to purchase organic food in emerging economies such as China, Brazil, and India. Nonetheless, some studies have suggested that domestic markets for organic food and products in emerging markets, such as China, have been increasing in the last decade [2]. Currently, there are more people that are willing to eat organic food as well as pay a premium price for it. This is mainly due to health concerns that are linked to inorganic food. Specifically, the demand for food quality is increasing in China, and the quality of food has become an essential component of food quality [3]. The change in attitude has mainly been influenced by recent health concerns in China. For instance, some of the health crises in China associated with food safety include: the baby milk incident, Avian Influenza (Bird Flu), and Spongiform Encephalopathy (Mad Cow disease). See a comprehensive review from the following book [3].

However, as has commonly been accepted by public, one of the major factors that is mainly associated with consumers’ willingness to pay for organic food is the price, due to the fact that such foods have commonly higher prices. Based on such thought, the main aim of this study is to critically investigate various research topics that are found in agriculture, economics, nutrition, marketing, and food journals addressing the factors that influence customers’ willingness to purchase organic food. The research will analyze the emergent but representative literature about Chinese consumers’ willingness to pay for organic foods. This will be achieved through a bibliometric mode of investigation. Therefore, the main aim of this study is to critically explore Chinese consumers’ perception of organic foods and their willingness to purchase organic food. The research question is: What are the major factors (addressed in the literature) that influence Chinese consumers’ willingness to pay (WTP) for organic food and products?

## 2. Methodology

Bibliometric studies are used with the theoretical perspective that the examination of citations enhances comprehension of the growth of contributions within a particular scientific field; it can identify when papers were written, and the relevance of such a publication in the currently. If a publication continues to be cited over time, historical value is assumed, making the source considered as a primary reference. Moreover, bibliometric identifies key topics contained in those keywords in the field. Progressive use of a keyword across numerous works and time indicates important areas, concepts, or topics in a field. If there is a significant change in keyword usage over some time, it is an indication that there is a paradigm shift. In such perspective, this study will assess journals and other scholarly works to identify the aspect that influences consumers’ willingness to pay for organic food and products in China. To our knowledge, there are not many published papers in the Social Science Citation Index, but it still has great potential because of its practical significance in the Chinese food market.

### 2.1. Data Selection

The selection of data from an extensive search of prior research in the field of consumer behavior, and specifically on the Chinese consumers’ willingness to pay for organic foods was an important primary step in answering the research question, and framing the current study in the context of the wide research in the field. The first step in search of previous research involved checking through various databases such as Blackwell, Proquest, SSRN, and AgEcon Search. These databases were selected as they have a large pool of resources, with both published works and also working papers that are relevant in answering the research question under consideration. Moreover, most of the works published in these journals are original papers whose research has been carried out either quantitatively or qualitatively.

The researcher identified the keyword queries for the different sub-areas of Chinese consumer behavior in reference to their willingness to pay for organic foods. The keywords associated with purchase of organic foods by the Chinese are specific to that area, and are important in ensuring that all factors are explored, and the emergent themes from the analysis are exhaustive. The keywords used to address all the subareas of the research questions included; ‘Chinese organic foods’, ‘willingness to pay’, ‘organic foods purchase’, organic foods purchase intentions’, ‘re-purchase intentions’, and ‘Chinese consumer behavior.’ These keywords were used comprehensively for purposes of covering the multidimensionality of the Chinese consumers’ behavior towards the purchase of organic foods. The search was intended to collect a large representative pool of original research that could allow this research to draw informed and fundamental conclusions.

Given that thousands of article results retrieved from the databases did not provide useful estimates and variables related to ‘willingness to buy’ organic foods among Chinese consumers, the researcher set a criterion that limited the scope to articles that precisely addressed the research problem and the research population. First, articles selected covering the concept of ‘willingness to pay’ were limited to those that focused on organic foods only. Other articles focusing on other aspects of food safety and sustainability such as pest-reduced foods, pesticide-free foods, and labeled foods were eliminated from this study. However, articles that focused on specific organic foods such as organic rice and organic fruits were included.

The concept of currency was also considered during the selection of the articles. Only articles published within the last five years were included in the study, limiting the articles only for those published between 2013 and 2018. The rationale for the time limit is derived from the fact that five years is a critical time in behavioral fields where adequate changes in customer needs and preferences can undergo significant changes. According to Simões [4], Chinese consumers, especially mature citizens, are very conscious about issues related to sustainability, and their behavior continues to change in alignment with their view of sustainable development. Since organic foods are related to sustainability in the long run, this time period was deemed appropriate in ensuring that the study reflected the current studies about Chinese consumer behavior in reference to the willingness to pay for organic foods.

The selected materials were also limited to Chinese consumers only. Most of the retrieved articles yielded results on willingness to pay for organic foods from consumers in other Asian countries, especially Thailand, India, Indonesia, Taiwan, and Malaysia among others. Since the inclusion of different Asian countries expanded the scope of the current study, only studies that were conducted from the perspective of the Chinese consumer were considered relevant for inclusion in the research.

### 2.2. Data Collection Procedure

The data collection process was conducted in stages. First, the primary search terms were used in each database one at a time. Then, the keywords were combined to form multiple phrases that refined the search and resulted in a wide range of results. The same procedure was repeated for all the databases until all the relevant articles had been retrieved. Articles were included if they meet the aforementioned inclusion criteria, otherwise the rest were not included. Only quantitative, qualitative, and systematic reviews that were peer-reviewed prior to publication were considered. Others such as newspaper articles and general commentary in economics were excluded. The study only utilized published works.

During the first stage of search, a total of 164 articles were identified as containing relevant information on the field of consumer behaviour, and especially the willingness to pay for organic foods. Due to the large body of previous research available for review and the broad scope of their literature, the inclusion criteria already identified was used to narrow the search. The abstract was used to refine the search and determine whether or not the article contained the required information. As such, all the articles that did not have an abstract were automatically excluded.

**Figure 1:**
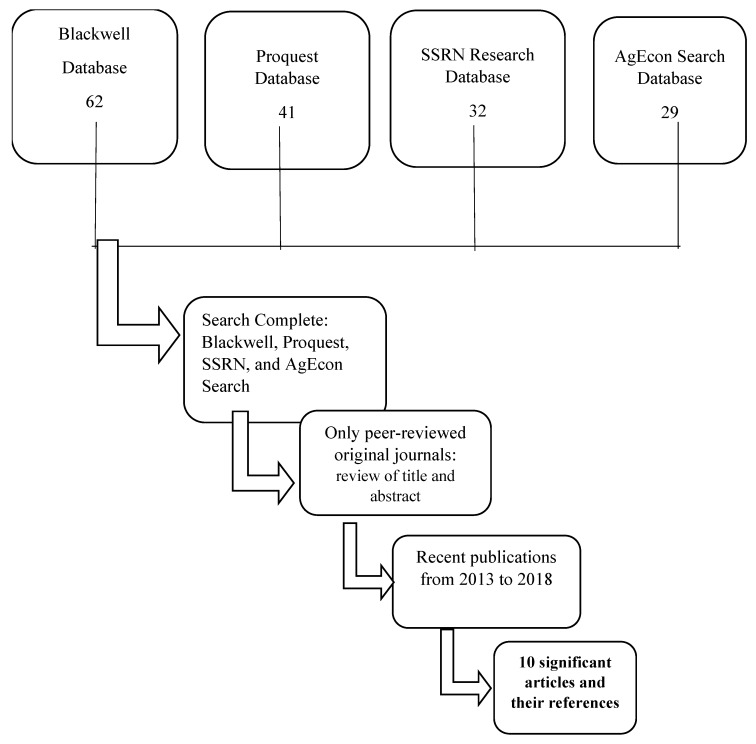
Overview of the sampling procedure.

After the review of the abstract for relevant information, the sample of articles selected was reduced to 67 articles. Broad categories were used to eliminate policy papers and other articles that did not specifically address the issue of organic foods from the perspective of Chinese consumers. Reviewing the articles to only include those published about Chinese consumers and those written in English, or those that have a translated version, narrowed the search to 43 articles. The final exclusion criterion was the time factor, which limited the sample to 10 articles. Figure 1 below demonstrates the sampling process and the narrowing of the number of articles from 164 to 10.

## 3. Results and Discussions

### 3.1. Bibliometric Matrix Synthesis

The results of the analysis of the selected articles based on the author, year of publication, title, methods, and results are presented in the Table 1 below. This analysis was important in performing the bibliometric analysis of the characteristics of selected studies and their contribution to the study.

### 3.2. Most Cited Keywords

**Figure 2:**
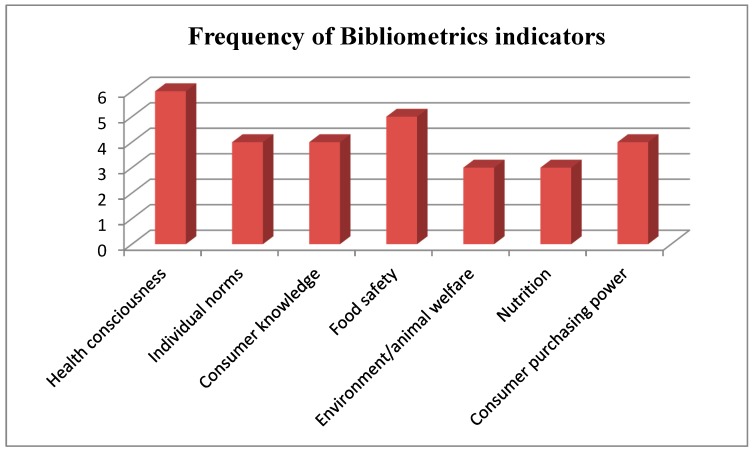
Keywords that were most cited.

The analysis of the articles revealed intersecting themes including health consciousness, individual norms, consumer knowledge, food safety, environment/animal welfare, and nutritional factors. Some articles reported multiple themes. Figure 2 below presents the major themes emerging from the keywords most cited in the selected articles. Due to space in the figure, please find the caption’s full description for each category in the main text.

This figure has demonstrated that the major concerns for the Chinese consumers’ WTP include the following.

#### 3.2.1. Health Consciousness

The result from the materials disclosed in the sampled literature showed that the more conscious the Chinese consumers are about their health, the more their willingness to pay for organic foods. As aforementioned, the Chinese people have a positive attitude towards organic foods due to the understanding that organic foods are good for health, as opposed to the conventional counterparts that are chemical and toxin-laden. Yang, Al-Shaaban, and Nguyen [12] conducted a study in which health consciousness was depicted as the primary factor influencing the purchase intention of consumers towards organic foods. The study found that organic foods are preferred since they are often perceived by consumers to be relatively free from chemicals, and may thus be thought of as safer for consumption. As such, they are more willing to buy the types of foods they are sure to consume without suspicion or worries.

According to Gan et al. [15], Chinese consumers have become increasingly aware of health concerns in the country’s food industry in relation to their general wellbeing. This notion was strongly held by the respondents in the study who demonstrated concerns about the quality of the water they are drinking and the foods they are consuming. The respondents also indicated that they constantly worrying about the conventional foods they eat due to the harmful chemicals and pesticides used to grow them. Others reported that were regular readers of health-related articles in books, magazines, and also in the newspaper. They also read the ingredients labels before making any purchase decisions to ensure that they do not pay for foods that have high levels of preservatives and additives. Gan et al. [15] attribute these tendencies to the rise in the number of health-conscious people in China, who demonstrate preferences for organic foods, hence a high level of willingness to pay for organic foods as they are aligned with their lifestyle choices. The study also notes that households with a history of consumption of organic food have fewer cases of chronic illnesses.

#### 3.2.2. Individual Norms

Many of the sampled studies have argued that individuals whose personal norms and attitudes are aligned with a positive attitude towards organic food products are more likely to demonstrate willingness to pay for organic foods compared to those who do not feel that paying for organic foods is the right thing to do. For example, Yang, Al-Shaaban, and Nguyen [12] found that Chinese consumers who feel they make a conscious choice to pay for organic foods, as well as those who believe that choosing organic foods is the right decision, are more willing to buy organic foods because their motivation is intrinsic and based on their attitudes.

In another study, Fang and Levy [5] found that there was a statistically significant relationship between the attitude of Chinese consumers towards organic foods and their purchase intentions. In this case, the study notes that the attitudes of the consumers are aligned with their personal norms, and there is a link between organic foods and the achievement of various values in life. The lifestyle of individuals is important in determining the values they give organic food products. If the value of organic foods is highly aligned with the achievement of life values, then Chinese consumers are highly likely to pay for the organic foods. In most cases, consumers who have a tendency to pay more attention to the foods they buy are more likely to pay for organic foods.

Yen [13] also provided valuable insight into this body of evidence by positing that Chinese consumers’ willingness to pay for organic foods is influenced by self-congruity and the need to self-identify with the social self-concept. Respondents indicated that eating organic foods fits well with their image of self, is consistent with the way they view themselves, and has helped them reflect on who they are at a personal level.

#### 3.2.3. Consumer Knowledge

Bibliometric-based evidence from this analysis demonstrates that Chinese consumers are more willing to pay for organic foods when they have sufficient awareness about the knowledge of organic foods, the benefits of organic foods, the nutritional values, and health impact in the long-run. For instance, Gan et al. [6] also reported that the level of knowledge of the consumers regarding organic foods is related to their willingness to buy organic food products. The positive correlation confirms the notion that consumers who have a high level of knowledge regarding the benefits of purchasing and consuming organic foods are highly likely to pay for organic foods. Gan et al. [6] explain that since organic foods are credence goods, information is critical in influencing consumers to buy organic foods as opposed to other conventional food products that are readily available, cheaper, and at the convenience of the consumer. The results of this study are also consistent with Yang, Al-Shaaban, and Nguyen [12] to the extent that limited consumer knowledge is a key impediment to the willingness of consumers to pay for organic food products. As such, it is essential that marketers of organic foods take into account the knowledge level of the market, understanding the concept of organic foods and their many benefits. The more people become knowledgeable about organic foods, the more their willingness to pay for them.

Similarly, Liu, Pieniak, and Verbeke [8] found that among safe foods, green foods, organic foods, and hazard-free foods, organic foods are the less known among the Chinese consumers due to the limited efforts to enhance consumer awareness. The study found that only a quarter of the respondents were aware of organic foods. Knowledge level of a consumer is a primary factor in forming a positive attitude towards the purchase of organic foods.

#### 3.2.4. Food Safety

With the increased cases of scandals in the Chinese food system, food safety has become a major factor to consider when making purchase decisions, especially for consumables. McCarthy [9] noted that most of the food safety issues are attributed to the high levels of additives and pesticides especially in genetically modified foods.

Gan et al. [6] also support the notion that food safety is a major contributor of consumers’ willingness to pay for organic food products. Health and food safety factors are important considerations that influence consumers to make purchase decisions in favor of organic foods. Organic foods are more nutritious in terms of vitamins and minerals, and they are also safe from pesticides, toxins, preservatives, and additives. With the increase in cases of food scandals in China, most consumers have become increasingly conscious about the safety of conventionally grown foods. The study also notes that there is limited information about food safety in China, which limits the extent to which consumers can make informed decisions. Increased awareness of the level of safety of foods in the Chinese markets can play a critical role in educating people to make informed health and nutritional choices that are good for their health.

According to Li and Xin [7], young Chinese consumers are motivated to pay for organic vegetables and fruits especially due to the recent food scandals in the country. Li and Xin [7] attribute the increasing demand for organic foods among Chinese consumers to the poor record of the country’s food system, which has increased the desire of the people to demand for health and safe foods that are free from contaminants and other disease-causing elements. The study explores various scandals related to conventional food products from counterfeit eggs, gutter cooking oils to milk and baby formula adulterated with melamine. These cases are just an isolated few that raise food safety concerns, giving organic food marketers an edge in terms of the influence to meet consumer needs and expectations of safe foods.

Xie et al. [10] found that most Chinese consumers are highly willing to pay for organic foods because they are assured that the safety of the food is guaranteed. Organic foods are grown without the use of chemicals and pesticides that may have traces in the end products that is taken to the consumers’ plates. A respondent was noted saying that she had found worms in organic apples several times, but that was an assurance that the apple was organically grown without any pesticides used to prevent the infection of the fruits by the worms. Another respondent also explained that she had previously worked in an organic certification body and she was aware of animals that were pumped with hormones and medicine, which was not the case in organically grown animals. As such, consumers are highly willing to pay for foods that are organically grown as that assures them of the safety of their foods.

In another study, Yang, Al-Shaaban, and Nguyen [12] also found that food safety was an important consideration in the willingness to pay for organic foods. Organic foods are safe for human consumptions as they are free from chemicals and other harmful substances that would result in ill health. Moreover, organic foods give consumers the confidence that they can consume the foods without fear or suspicion.

#### 3.2.5. Environment/Animal Welfare

The results of this study indicate that environmental and animal welfare concerns are related to a high willingness of Chinese consumers to pay for organic food products. A study conducted by Xie et al. [10] found that the majority of Chinese consumers are ethically minded when making consumption decisions, based on the increased cases of food fraud in the industry. Such consumers have a greater willingness to purchase organic foods as they are aligned with personal objectives of preserving the integrity, beauty, and stability of their households, and also the environment. A respondent in the study stated that the environment in which they live is free from plastic waste, pesticide bottles in the lands, streams and rivers, and it is such a beautiful place that they would wish it for the next generation. The respondents acknowledged the high costs of organic foods, noting that they would rather eat less and preserve their health, the environment, and the welfare of animals than eat more conventional foods.

McCarthy [9] also found that Chinese consumers have a positive attitude towards the ethical principles that guide organic foods farming, including the care for the welfare of the animals. Despite the fact that health concerns outweigh the environmental concerns when making purchase decisions for organic foods, McCarthy [9] notes that consumers are more motivated to pay for organic foods that are linked to moral attitudes, the type of decisions that make people feel that they are doing the right thing for themselves, and for the environment. In this case, consumers are more likely to pay for organic products that are farmed under ethical and moral foundations, and those that ensure environmental conservation and care for the welfare of animals.

According to Zhu [16], the ecological motive has a significant influence on the intention of people to purchase organic foods, which then translates into their willingness to pay for organic foods. The study combines both environmental concerns and animal welfare into the ecological motive, noting that these characteristics are important for organic food consumers, as they are major determinants of whether or not consumers will pay for the organic foods as opposed to the conventionally grown foods. Zhu [14] cites several studies to affirm this conclusion by noting that previous research supports the results in terms of the positive ethical role that influences the purchase decisions of organic food products. The study also explains that organic foods consumers have a high sense of consciousness towards the environmental, and that is their major motivation to buy organic foods. This variable is also related to the self-identity variable, as individuals make the purchase decisions based on their belief systems of doing the right thing.

#### 3.2.6. Nutrition

Nutrition is a critical driver for the purchase of organic foods for Chinese consumers. Xie et al. [10] found that consumers of organic foods are reported to value organic foods because organic foods are laden with nutritional values compared to conventionally grown food products. A total of 65% of respondents in the study reported that they buy organic foods because organic foods are fresher and have a better taste. As such, Chinese consumers are more willing to pay for organic foods if they perceive that the food has more nutritional value compared to the other types of foods that are not fresher, healthier, and rural-like.

The findings in Xie et al. [10] are consistent with the results of the study conducted by Li and Xin [7]. The study found that people reported that organic foods are more nutritious, have a better taste, and are safer and good for human consumption. The labeling of organic food products is also important in providing transparent information that shape the consumers’ positive attitudes towards the purchase of organic foods. Li and Xin [7] interpret the finding of the study in the content of a wider body of literature and note that other studies found organic foods to have a high level of minerals such as magnesium, iron, phosphorous, and vitamins such as Vitamin C. These foods are also very nutritious, hence the positive attitude of Chinese consumers towards the purchase of organic foods.

#### 3.2.7. Consumer Purchasing Power

The results of this analysis demonstrate that there is a positive relationship between the consumers’ purchasing power and their willingness to pay for organic foods. Gan et al. [6] found that organic foods are expensive compared to conventional foods that are readily available. People with a high social standing are more likely to purchase organic foods as social markers. Education is also attributed to the increase in purchasing power of consumers in China as they have high incomes compared to their uneducated counterparts. Higher income households demonstrate a higher willingness to pay for organic foods because they have the ability to pay for a premium to derive the benefits of organic foods. They believe that organic foods are more preferable, nutritious, healthier, and more aligned with their social values as opposed to the conventional alternatives. On the other hand, price is also discriminative in the sense that consumers who want to consume organic foods cannot do so because of the limited resources. Gan et al. [6] notes that the level of price sensitivity among Chinese consumers is very high, such that people make purchase choices that give them value for money. In this case, they are less willing to pay a premium to get organic foods when conventional foods are cheaper and highly convenient.

The results of Gan et al. [6] are also confirmed by findings reported by Xie et al. [10] in which the respondents reported that the high price for organic foods was a major deterrent to the purchase of organic foods, especially for young Chinese consumers. In Nanjing and Shanghai, the difference in the cost of organic and conventional fruits and vegetables was found to be very high for the average consumers to afford. Some respondents also indicated that while they understood the manual work and the high cost of organic farming compared to conventional farming, the premium price charged for organic farming made it very costly that most people could not buy. Nevertheless, the study found that most people regarded the cost of organic foods as necessary, hence a high willingness to purchase.

Xu, Su, and Lone [11] also found that price is a major determinant of the willingness of Chinese consumers to pay for organic foods. The results of the study support the premise that low food expense consumers are attracted to brands of rice that are affordable, hence low willingness to pay for organic foods. On the other hand, high expense consumers are willing to pay a premium price for organic rice.

### 3.3. Nature of Evidence

The majority of the studies were made of quantitative studies while the rest of the studies were comprised of mixed methods and systematic reviews.

Such results show an urgent call for utilization of more diverse methodologies, in order to broaden the possible scopes of findings. Quantitative investigation for the issue of consumers’ WTP might be more suitable for mature/established research questions, while other methods (especially qualitative methods) might benefit from innovative explorations and thus could facilitate the growth of the literature.

## 4. Conclusions

The purpose of this bibliometric study was to understand the knowledge structure of scientific literature on Chinese consumers’ WTP for organic foods. Due to the fact that there is limited research on the WTP for organic food in China, the bibliometric methodology was vital in gathering information that is likely to influence Chinese consumers’ willingness to pay for organic food. The research question was answered with the findings. What are the major factors (addressed in the literature) that influence Chinese consumers’ WTP for organic foods? The first relates to health consciousness, which reminds that modern consumers should be vigil regarding foods that can harm their health. The bibliographic analysis also indicated individual norms, consumer knowledge, food safety, environmental concerns, animal welfare, and purchasing power to be very important aspects that influence consumers’ WTP in China.

Despite the various perceptions that are related to organic food, the purchasing power remains the strongest fact that should be considered when purchasing organic food [1,10]. As deduced from the study, it is clear that the level of consumption of organic foods in China is positively correlated to the income levels of the consumers. Despite the fact that most consumers may be aware of the health benefits of consuming organic foods, they do not purchase them because they are expensive compared to conventional foods that are cheaper and readily available. Although the various attributes identified can prompt a Chinese consumer to pay a premium price for organic foods, it is very important that advocates and marketers of organic foods consider the price. The price might be a major hindrance for the Chinese consumer to pay for organic foods. Nonetheless, it is evident that despite the premium price associated with organic foods, there are some consumers that are willing to pay more because of issues, such as the perceived health and nutritional benefits that are associated with organic foods. The nutritional value of organic foods is a major motivation that has a positive impact on influencing consumption patterns.

There are some limitations that might stimulate future research ideas. The goal of this paper was not to conduct a thorough check of the literature, but to draw attention and stimulate innovative and timely research thoughts by reviewing a small numbered, but critical and representative, set of papers (for a now arising market for organic foods). In such a sense, it makes sense to review the relevant articles in the past five years. However, the reviewed scope and number of papers could be completed for future studies in order to gain richer information.

## Figures and Tables

**Table 1:** Characteristics of the sampled studies.

No.	Author and Year of Publication	Title	Methods	Results
1	Fang and Levy [5]	‘An Analysis of Consumption and Purchasing toward Organic Fruits: Cross-Countries Study between China and France’	A quantitative research design was used to collect data from China and France. An online questionnaire survey utilizing a sample of 261 respondents was used.	The study reported that the Chinese and French consumers have a positive attitude towards the purchase of organic foods, in line with their respective planned behavior. Food-related lifestyles were found to be major drivers that influence purchase decisions.
2	Gan, Zhiyou, Tran, Cohen and Xiangxiang [6]	‘Consumer attitudes towards the purchase of organic products in China’	A quantitative research design was adopted where 700 structured questionnaires were used to collect data from a sample in Kunming City. The data sought to measure the attitude of the respondents towards the purchase of organic food products sold in the market.	The findings from this research show household income as the major determinant of the willingness to purchase organic foods. This is because organic foods are expensive compared to conventional foods that are readily available. People with a high social standing are more likely to purchase organic foods as a social marker. Moreover, Chinese households need more information about the benefits of organic foods, especially due to the big scandals in a country of tainted food products.
3	Li and Xin [7]	‘Factors influencing organic food purchase of young Chinese consumers.’	This quantitative research used a survey questionnaire to gather data about the factors that influence the Chinese consumers’ consumption of organic foods, and their purchase intentions.	The main factor that influences Chinese consumers to purchase organic foods is food safety. Other factors include nutritional values, animal welfare, and environment-friendly. Young Chinese consumers are motivated to pay for organic vegetables and fruits especially due to the recent food scandals in the country.
4	Liu, Pieniak, and Verbeke [8]	‘Consumers’ attitudes and behaviour towards safe food in China: A review.’	This systematic review includes a sample of 34 studies that focused on the willingness of consumers to pay for safe foods. The article includes organic foods, green foods, and hazard-free foods.	The findings demonstrated that the Chinese consumers have a high level of awareness of safe foods, but low recognition of the concept of safe foods, including labels and identification of safe foods. Generally, there is a positive attitude towards the purchase of safe foods in the Chinese markets.
5	McCarthy [9].	‘Trends in organic and green food consumption in China: Opportunities and challenges for regional Australian exporters’	A quantitative research design was used to determine the willingness of consumers to pay for food safety, focusing on both organic and green foods. The sample of 250 certified organic and green foods consumers from Beijing, Shanghai, Guangdong, and Chongqing participated in the study.	The willingness of Chinese consumers to purchase organic foods is inspired by health and environmental factors. Moreover, most Chinese consumers who purchase organic foods regularly distrust the Chinese food system; hence their willingness to pay for organic foods is born out of food safety concerns.
6	Xie, Wang, Yang, Wang and Zhang [10]	‘Consumer perceptions and attitudes of organic food products in Eastern China.’	The study utilized a mixed methods research design combining both qualitative and quantitative data. The data was collected through a survey administered from consumers in Nanjing and Shanghai, east of China.	The major motivation for willingness to purchase organic foods for consumers in East China is driven by food safety and health concerns. Also, education level and higher purchasing power are attributed to increase the willingness of consumers to purchase organic foods. Lack of awareness and knowledge about the benefits of organic foods is a major barrier to willingness to purchase organic foods in China.
7	Xu, Su and Lone, [11]	‘Chinese consumers’ willingness to pay for rice.’	A quantitative study conducted using a survey administered in Chongqing and Chengdu, as they are the largest rice consumption cities in China. A conditional logit model was used to analyze the data.	Price is a major contributor of the purchase of organic rice in Chongqing and Chengdu. High demand for organic rice was reported in higher food expense consumers as they are willing to pay a premium for the organic rice.
8	Yang, Al-Shaaban and Nguyen, T. B. [12]	‘Consumer attitude and purchase intention towards organic food: A quantitative study of China.’	A quantitative research design was conducted through an online survey of Chinese consumers. The study was based on six hypotheses.	The results of the analysis indicated that the Chinese consumers’ willingness to pay for organic foods is influenced by health consciousness, individual norms, and consumer knowledge.
9	Yen [13]	Managing self-congruity to influence behavioral intention in organic food contexts in Fujian province, China.	This quantitative study employed 200 questionnaire surveys from organic food consumers in Fujian province.	The results of the analysis demonstrated that self-congruity has a major influence on the willingness to pay for organic foods, including aspects such as self-image, consistence with self-perception, self-reflection, and similar people purchasing organic foods. The ideal social concept should be a bridge between the consumers’ self-image and the image of organic foods.
10	Zhu [14].	‘Using the theory of planned behavior to investigate what influences Chinese intention to purchase organic food.’	A quantitative research design that utilized data collected from 216 in-depth surveys. The sample population was students from various universities in Hubei Province.	The study found that consumer identity and ecological motive were the biggest drivers of consumers’ willingness to purchase organic products. The study concluded that since people’s motivation to pay for organic foods is out of ecological concerns, and attitudes, marketers of organic foods should make such variables a priority by connecting organic foods to environmental values.
